# Influence of Quantitative Variables on Residency Match: A Retrospective Cohort Analysis

**DOI:** 10.7759/cureus.100385

**Published:** 2025-12-30

**Authors:** Bhagvat J Maheta, Ashley Niu, Ramy Khalil, Priya Manhas Yun, Megan D Hsu, Muhammad Karabala, Caroline Goswami, Jose L Puglisi, Jimmy Wen, Eldo E Frezza

**Affiliations:** 1 Surgery, Northwestern University Feinberg School of Medicine, Chicago, USA; 2 Surgery, California Northstate University College of Medicine, Elk Grove, USA; 3 Pediatrics, California Northstate University College of Medicine, Elk Grove, USA; 4 Medicine, California Northstate University College of Medicine, Elk Grove, USA; 5 Internal Medicine, California Northstate University College of Medicine, Elk Grove, USA; 6 Internal Medicine, California Pacific Medical Center, San Francisco, USA; 7 Biostatistics, California Northstate University College of Medicine, Elk Grove, USA; 8 Physical Medicine and Rehabilitation, California Northstate University College of Medicine, Elk Grove, USA

**Keywords:** accreditation council for graduate medical education (acgme), competitive specialties, probability, quantitative study, residency match

## Abstract

Introduction

The residency match process entails a comprehensive evaluation of each medical student applicant, and multiple aspects of the application affect a student's competitiveness for their chosen specialty. Quantitative variables associated with the match application were selected for analysis, as they are the most objective and consistently reported metrics. This study aims to identify the quantitative variables that are most strongly correlated with determining residency match probability.

Methods

This was a retrospective cohort study conducted between 2013 and 2021, utilizing data from the Association of American Medical Colleges Report on Residents. Possible predictors of matching, including average United States Medical Licensing Examination (USMLE) Step 1 and Step 2 scores, number of research experiences, number of research abstracts, presentations, and publications, number of volunteer experiences, and number of work experiences, were analyzed over time for each Accreditation Council for Graduate Medical Education (ACGME)-accredited specialty using percent variation. Radar plots were created to determine the most influential categories in determining the probability of matching for each specialty.

Results

Overall, research abstracts, presentations, and publications had the highest association with an applicant's probability of matching into their selected specialty. Standardized test scores were most positively associated with successful matches into Otolaryngology, Orthopedic Surgery, Plastic Surgery, and Neurological Surgery, with high Z scores (1.69, 1.68, 1.62, and 1.52, respectively, for Step 1).

Conclusion

A thorough analysis of the available objective data suggests that scholarly activities, including research abstracts, presentations, and publications, are the single most important quantitative variable influencing a student’s likelihood of matching. Future research can explore understanding the essential role of qualitative variables, such as letters of recommendation and personal statements.

## Introduction

In 1992, there were more applicants to residency programs than the total available positions [1]. This trend has continued, and in 2022, 42,459 medical graduates competed for 36,277 postgraduate year 1 (PGY-1) residency positions [1]. Within more competitive residency specialties, it has become increasingly difficult for an applicant to match; the elements of a successful application have evolved significantly over the years, and variations exist among specialties in terms of factors that make an applicant competitive [1,2].

Across the published literature, the aspects of successful residency applications differ, particularly across specialties [3-6]. Furthermore, the benchmarks of academic success and research productivity continue to increase. DePasse et al. explain that, among matched orthopedic surgery applicants between 2007 and 2014, the average United States Medical Licensing Examination (USMLE) Step 1 and USMLE Step 2 scores increased significantly, from 234 to 245 and 235 to 251, respectively [7]. The trend of increasing competitiveness has profoundly affected how medical students approach the match; Williamson et al. state that the average medical student in 2020 submitted 70 applications to residency programs, with senior MD students submitting an average of 96 applications for orthopedic surgery, a number that has been increasing [1,8].

A wide range of variables can impact a student’s success in the annual match, and students may sometimes be left unsure of where to focus their efforts within their preclinical and clinical years. Subjective metrics, such as personal statements and letters of recommendation (LORs), are crucial to the evaluation of a candidate; a survey of Emergency Medicine program directors found that LORs from emergency medicine physicians were the second most commonly cited factor used to decide whether or not to interview a candidate [9]. However, within the scope of quantitative evaluation, a certain degree of variability exists across specialties in terms of “important” predictors of a successful match. Therefore, it remains inconclusive which factors of the residency application are influential in a program’s decision regarding an applicant.

To build upon previous literature, we specifically considered only numerically reported (quantitative) variables in the residency match process, as they are objective metrics that can be statistically analyzed. This study aims to analyze the influence of these quantitative variables within applications into all Accreditation Council for Graduate Medical Education (ACGME)-accredited medical specialties over the past nine years. Through a retrospective cohort analysis, this study assesses the relative importance of surveyable variables on matching into each specialty, to better inform undergraduate medical students as they undergo medical school. Although a more thorough understanding of the most important predictors for matches into each specialty will not address the deficiency in residency positions across specialties, this study will allow future applicants to focus their efforts toward optimizing their extracurriculars for their specialty of interest by clarifying which benchmarks make them competitive and thus increasing their likelihood of matching into their preferred specialties. The primary objective of this retrospective study was to analyze the association between quantitative residency application variables and residency match outcomes. Secondary objectives were to explore differences across medical specialties and trends over time in these variables.

This article was previously presented at the American College of Surgeons Clinical Congress in San Francisco on October 20, 2024.

## Materials and methods

Data collection

To evaluate how different quantitative variables of the residency application are associated with matches into ACGME accredited medical specialties, our team collected and analyzed applicant demographics, average USMLE Step 1 Examination scores, USMLE Step 2 Examination scores, number of research experiences, number of research abstracts, presentations, and publications, number of volunteer experiences, and number of work experiences of matched applicants into each medical specialty from 2013 to 2021. The study population included residents who matched into an ACGME-accredited specialty between the years of 2013 and 2021 (n = 324,757), including those who graduated from MD or DO schools in the United States, as well as international medical graduates (IMGs). This data was collected from the publicly available Association of American Medical Colleges (AAMC) Report on Residents, which outlines the medical school statistics/ experiences of first-year residents across the nation by medical specialty, who just completed the match process. The purpose of this report is to provide standardized and comprehensive information on residency applicants in the United States. Additionally, past year reports were requested from the AAMC to obtain comprehensive data for all U.S. residents who applied through the match process for residency applications. To maintain the generalizability of the data analyzed in this study, sub-reports from the AAMC Report on Residents were not utilized. The average of the quantitative variables for each specialty was provided by the AAMC Report on Residents. The variables collected and analyzed in this study encompass all of the quantitative variables that were available through the AAMC database and could be statistically analyzed. Thus, no imputation, exclusion, or substitution procedures for data were required as no missing data were present for these variables analyzed. Data extraction and processing were verified by a second independent reviewer to ensure accuracy.

Data analysis

All of the data were comprehensively analyzed statistically by a statistician (JP). Variability over time in various quantitative variables, including average USMLE Step 1 Examination score, USMLE Step 2 Examination score, number of research experiences, number of research abstracts, presentations and publications, number of volunteer experiences, and number of work experiences for each ACGME-accredited specialty, was analyzed using the statistical software GraphPad Prism v10.1.1 (GraphPad Software, San Diego, CA, USA). The mean and standard deviation for each of the quantitative variables were calculated for each year from 2013 to 2021. The Z score provides a standardized score for each quantitative variable between specialties, indicating how many standard deviations away a certain specialty is from the average. A positive Z score indicates that a certain specialty is above the average for the specific quantitative variable, whereas a negative Z score indicates that the specialty is below the average. The radar plot outlines all of the quantitative variables important for matching into each specialty. These plots were created using Z scores for each quantitative variable on equidistant radial axes using GraphPad Prism. The dotted line indicates the average across all specialties, and the solid line indicates the average Z score of matched applicants into that specialty across multiple variables; if the solid line is above the dotted line, applicants who matched into that specialty were above average in that specific quantitative variable. Radar plots were created to visually summarize the standardized specialty-level metrics.

## Results

Overall demographics

From 2013 to 2023, 324,757 applicants successfully matched into a specialty, with 38.57% (125,259/324,757) of those being women (Table 1).

**Table 1:** Number of Overall Demographics of Matched Applicants and Average Percentage of Women by Specialty (2013-2023)

Specialty	Number of Matched Applicants	Average % Women
Anesthesiology	13,819 (4.26%)	29.47%
Child Neurology	1,389 (0.43%)	55.36%
Dermatology	295 (0.09%)	58.54%
Emergency Medicine	23,620 (7.27%)	31.78%
Emergency Medicine-Anesthesiology	7 (0.002%)	-
Emergency Medicine-Family Medicine	55 (0.02%)	-
Family Medicine	40,312 (12.41%)	36.26%
Family Medicine-Preventative Health	42 (0.01%)	-
Internal Medicine	83,915 (25.84%)	21.13%
Medicine-Anesthesiology	44 (0.01%)	45.65%
Medicine-Dermatology	72 (0.02%)	58.48%
Medicine-Emergency Medicine	307 (0.09%)	31.25%
Medicine Genetics	21 (0.006%)	30.08%
Medicine-Pediatrics	4,150 (1.28%)	53.89%
Medicine Preliminary	19,256 (5.93%)	26.31%
Medicine-Preventative Health	72 (0.02%)	33%
Medicine Primary	4,156 (1.28%)	26.31%
Medicine-Psychiatry	244 (0.08%)	39.81%
Interventional Radiology	273 (0.08%)	20.50%
Neurology Disability	37 (0.01%)	-
Neurological Surgery	2,451 (0.75%)	17.26%
Neurology	6,137 (1.89%)	28.20%
OBGYN	14,882 (4.58%)	72.59%
OBGYN Preliminary	110 (0.03%)	-
Orthopedic Surgery	8,505 (2.62%)	14.81%
Osteo Neuromuscular	35 (0.01%)	-
Otolaryngology	3,537 (1.09%)	35.33%
Pathology	6,309 (1.94%)	23.59%
Pediatrics	30,177 (9.29%)	49.80%
Peds-Anes	80 (0.02%)	62.85%
Peds-Emergency Medicine	91 (0.03%)	51.83%
Peds-Genetics	185 (0.06%)	47.00%
Peds-PMR	32 (0.01%)	56.85%
Peds Preliminary	267 (0.08%)	-
Peds Primary	863 (0.27%)	-
Peds-Child Psych	224 (0.07%)	71.00%
Physical Medicine Rehabilitation	1,498 (0.46%)	30.10%
Plastic Surgery	1,804 (0.56%)	40.53%
Psychiatry	17,999 (5.54%)	33.80%
Psych (Categorical)	119 (0.04%)	62.49%
Psych Neuro	32 (0.01%)	37.64%
Radiology Oncology	155 (0.05%)	28.62%
Diagnostic Radiology	1,408 (0.43%)	22.08%
Surgery	14,881 (4.58%)	-
Surgery Prelim	8,015 (2.47%)	-
Thoracic Surgery	417 (0.13%)	19.86%
Transitional	11,747 (3.62%)	29.24%
Vascular Surgery	711 (0.22%)	32.20%
Overall	324,757	38.57%

**Figure 1:**
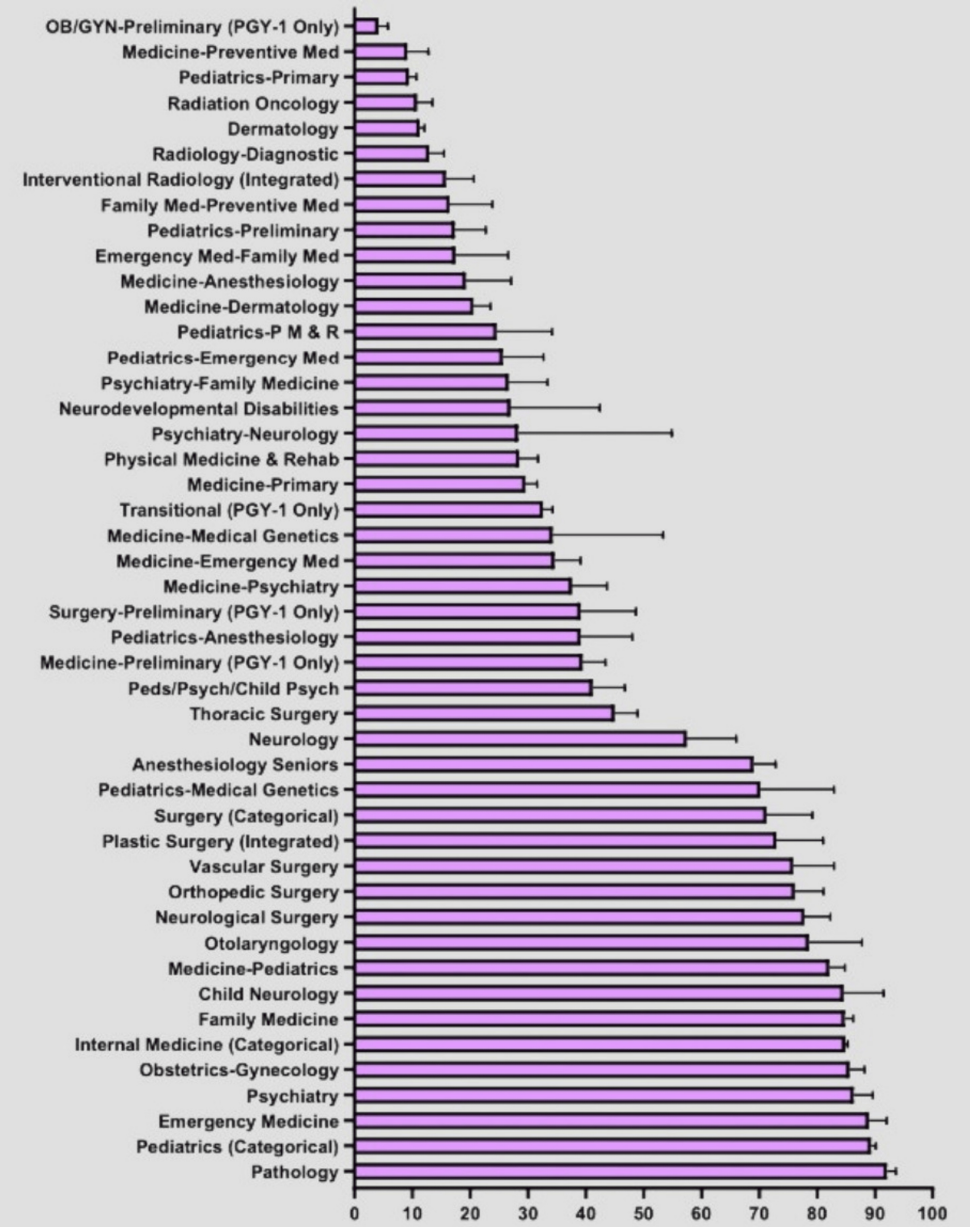
Percentage of Matched Senior Applicants in Each Specialty

**Figure 2:**
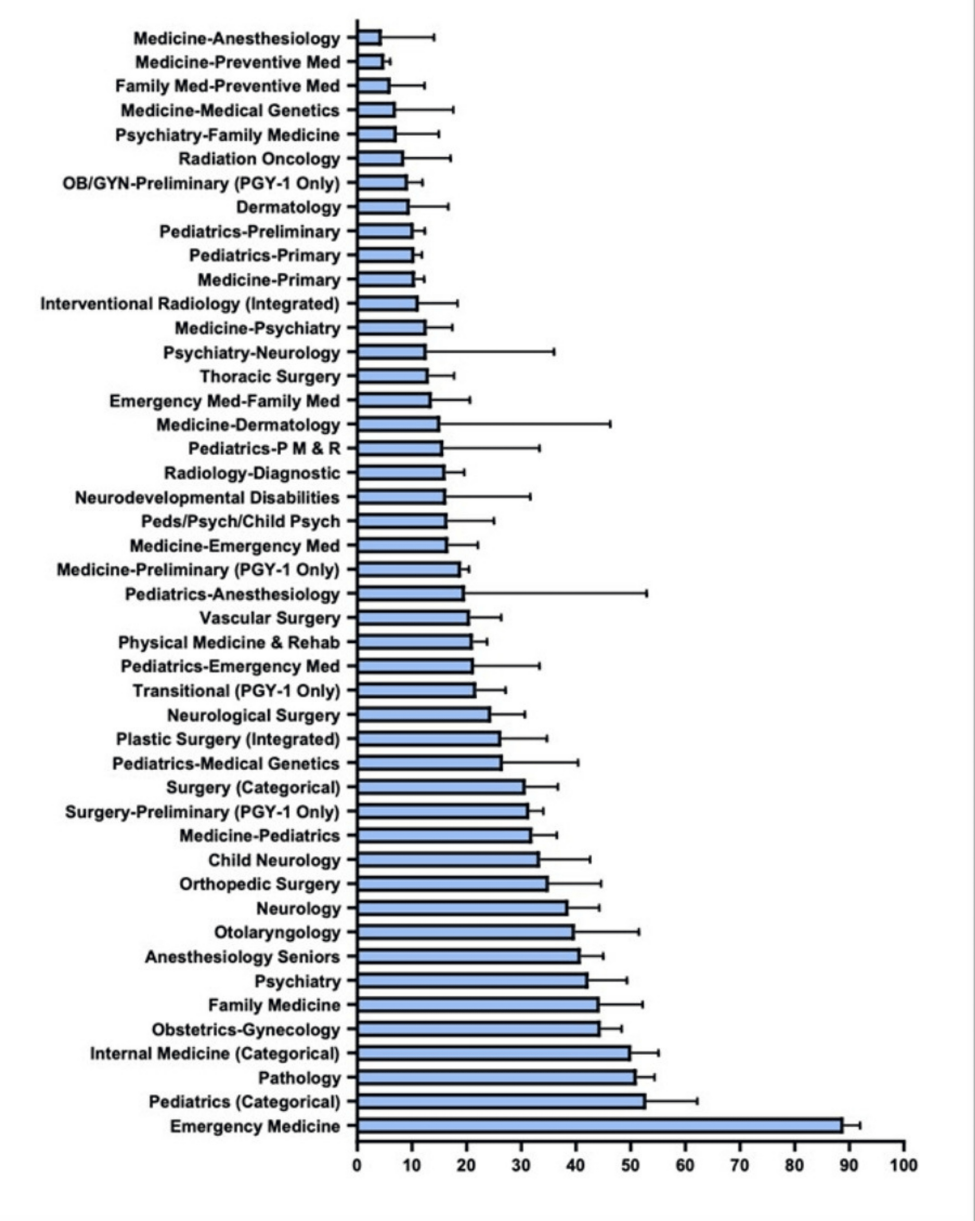
Percentage of Matched Non-senior Applicants in Each Specialty

The specialties with the highest number of matched applicants include internal medicine, with 83,915; family medicine, with 40,312; and pediatrics, with 30,177. The specialties with the lowest number of matched applicants include emergency medicine-anesthesiology, with 7; medical genetics, with 21; and psychiatry-neurology and pediatrics-physical medicine and rehabilitation, with 32. The specialties with the highest percentage of matched female applicants include obstetrics/gynecology (OBGYN) at 72.59%, pediatrics-child psychiatry at 71%, and pediatrics-anesthesiology at 62.85%. The specialties with the lowest percentage of female-matched applicants include orthopedic surgery at 14.81%, neurological surgery at 17.26%, and thoracic surgery at 19.86%. The percentages of senior MD students and non-senior MD students matching in each specialty were also analyzed (Figures 1-2).

Variations over time

**Figure 3:**
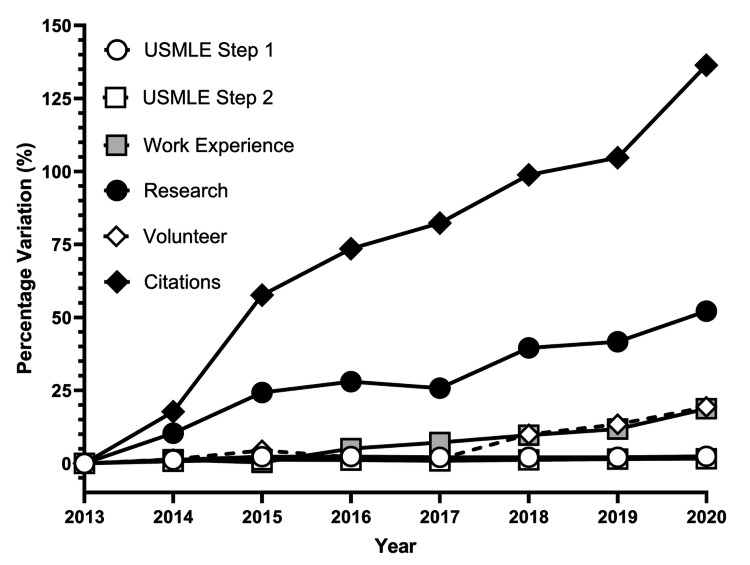
Percentage Variation of Matched Applicants’ Quantitative Characteristics Percentage variation of matched applicants’ quantitative indexes. Percentage values were calculated using 2013 as the baseline. United States Medical Licensing Examination (USMLE) Step 1 and Step 2 scores increased marginally (2.41% and 1.63% respectively), Work Experience and Volunteer Work augmented in a similar fashion (18.75% and 19.30% respectively). The number of Research Experiences climbed 52.13% and the number of Citations exhibited an increase of 136.36%.

From 2013 to 2020, the statistical analysis shows that the percentage variations on different quantitative variables, such as Step 1 score, Step 2 score, research experiences, research abstracts, presentations, publications, and volunteer experiences, were assessed for each specialty as seen in Figure 3.

Analysis revealed that both Step 1 and Step 2 scores remained consistent over time, with their curves overlapping. The number of research experiences exhibited a gradual rise throughout the years. Additionally, the number of research abstracts, presentations, and publications demonstrated a notable increase, surpassing the growth observed in research experiences. It was not until 2018 that the number of volunteer experiences began to show an upward trend.

Standardized test scores 

**Figure 4:**
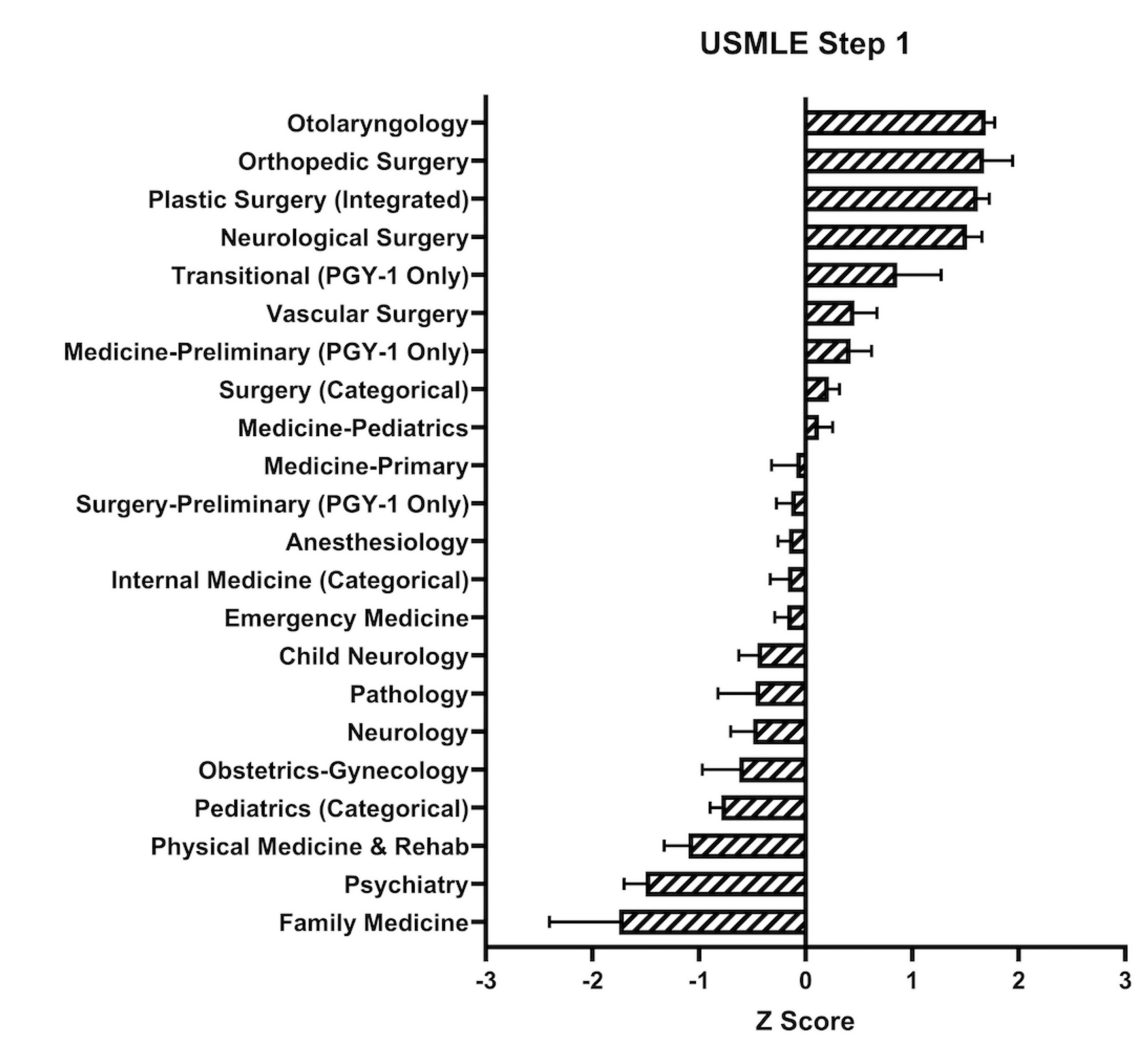
Matched Applicants’ Step 1 Scores by Specialty Matched applicants’ United States Medical Licensing Examination (USMLE) Step 1 scores were analyzed for each specialty. Average values and standard deviations were obtained from 2013 to 2020. Then, a Z score was calculated for each specialty. The top four specialties were Otolaryngology, Orthopedic Surgery, Plastic Surgery (Integrated), and Neurological Surgery.

The Z score for each specialty in Step 1 scores was calculated (Figure 4).

Otolaryngology, Orthopedic Surgery, Plastic Surgery, Neurological Surgery, and Transitional had the highest positive Z scores, at 1.69, 1.68, 1.62, 1.52, and 0.86, respectively. The highest negative Z scores included Family Medicine, Psychiatry, Physical Medicine & Rehab, Pediatrics, and OB/GYN, at -1.75, -1.50, -1.10, -0.79, and -0.62, respectively.

**Figure 5:**
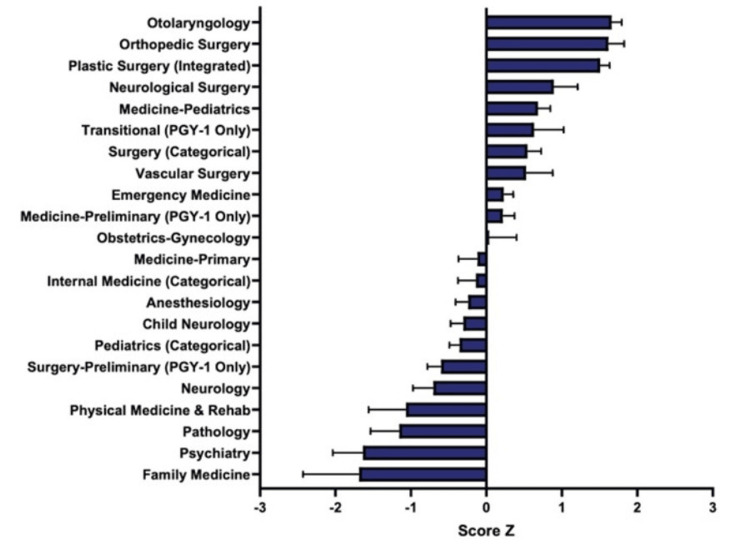
Matched Applicants’ Step 2 Scores in Each Specialty

The Z scores for individual specialties based on Step 2 scores were calculated (Figure 5).

The highest positive Z scores included Otolaryngology, Orthopedic Surgery, Plastic Surgery, Neurological Surgery, and Medicine-Pediatrics, at 1.66, 1.62, 1.51, 0.89, and 0.69, respectively. The highest negative Z scores included Family Medicine, Psychiatry, Pathology, Physical Medicine & Rehab, and Neurology, at -1.69, -1.64, -1.16, -1.07, and -0.70, respectively. Notably, OB/GYN had the lowest positive Z score, at 0.04.

Volunteer experiences

**Figure 6:**
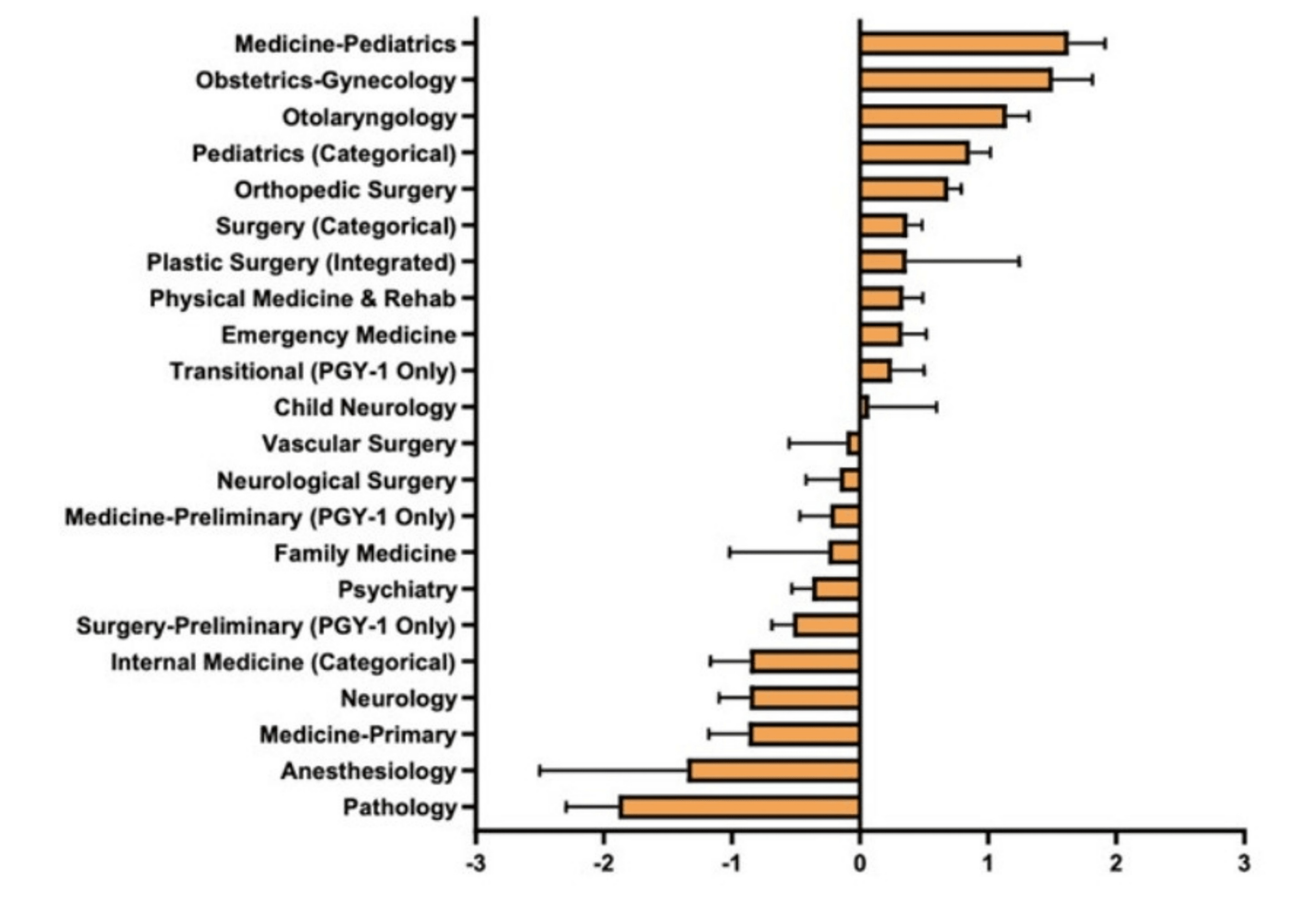
Matched Applicants’ Volunteer Experience in Each Specialty

The Z score for each specialty based on volunteer experiences was calculated (Figure 6).

The highest positive Z scores included Medicine-Pediatrics, OB/GYN, Otolaryngology, Pediatrics, and Orthopedic Surgery, at 1.63, 1.51, 1.15, 0.86, and 0.69, respectively. The highest negative Z scores included Pathology, Anesthesiology, Medicine-Primary, Neurology, and Internal Medicine, at -1.89, -1.35, -0.87, -0.86, and -0.86, respectively. Z scores closest to zero were found for Child Neurology, at 0.07, and Vascular Surgery, at -0.16. 

Work experiences

**Figure 7:**
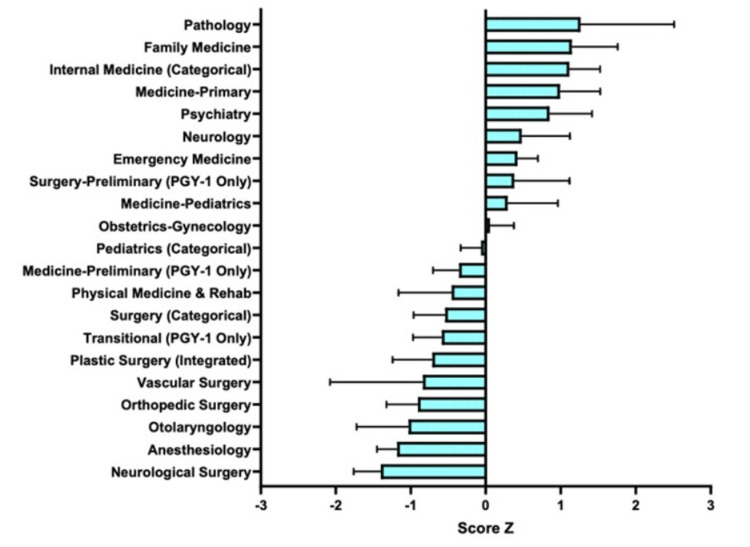
Matched Applicants’ Work Experience in Each Specialty

The Z score for each specialty in work experiences was calculated (Figure 7).

The highest positive Z scores included Pathology, Family Medicine, Internal Medicine, Medicine-Primary, and Psychiatry, at 1.265, 1.149, 1.114, 0.990, and 0.850, respectively. The highest negative Z scores included Neurological Surgery, Anesthesiology, Otolaryngology, Orthopedic Surgery, and Vascular Surgery, at -1.402, -1.182, -1.030, -0.907, and -0.839, respectively. Z scores closest to zero were found for OB/GYN, at 0.056, and Pediatrics, at -0.063.

Research experiences

**Figure 8:**
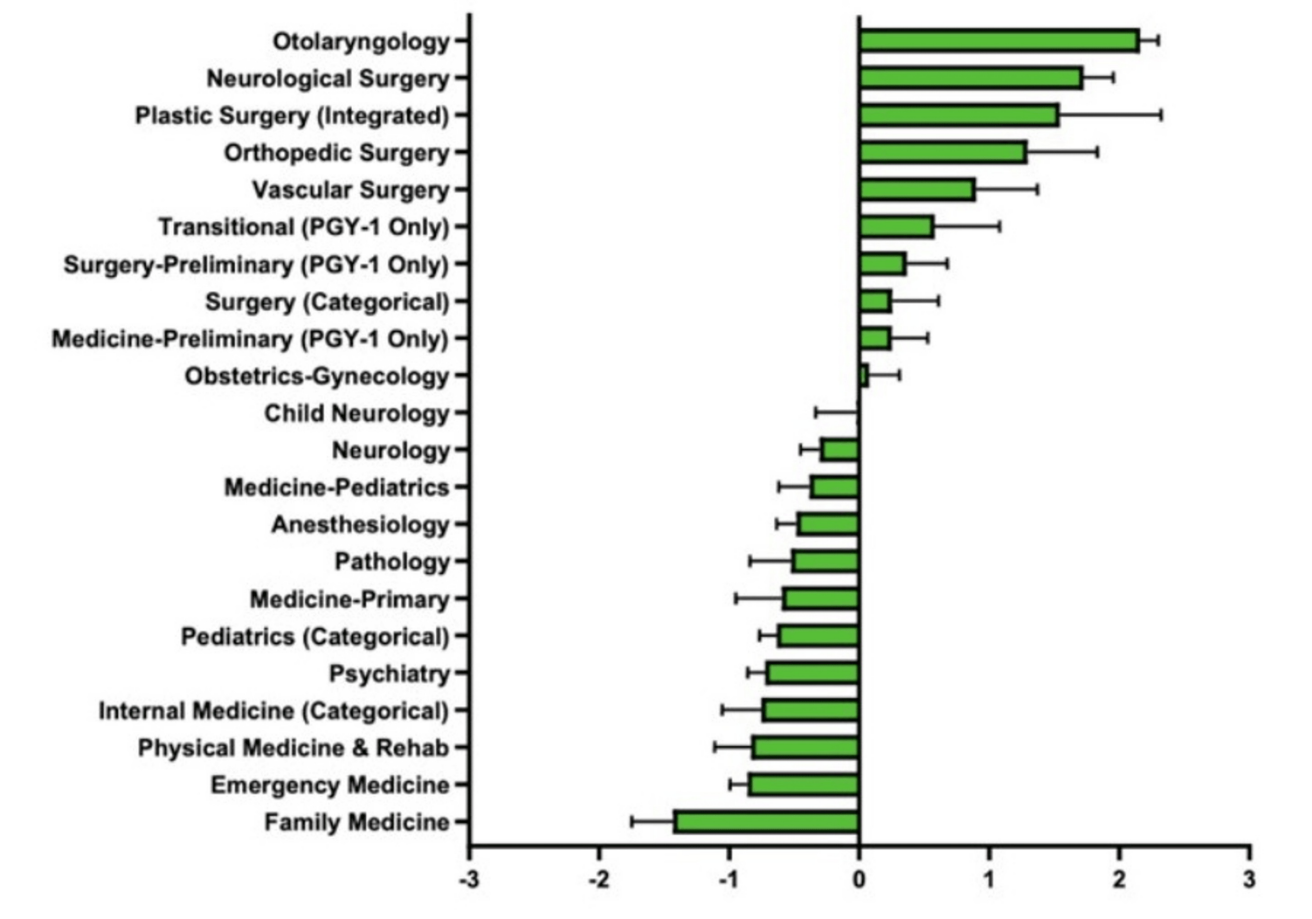
Matched Applicants’ Research Experience in Each Specialty

The Z score for each specialty in research experiences was calculated (Figure 8).

The highest positive Z scores included Otolaryngology, Neurological Surgery, Plastic Surgery, Orthopedic Surgery, and Vascular Surgery at 2.16, 1.73, 1.54, 1.30, and 0.90, respectively. The highest negative Z scores included Family Medicine, Emergency Medicine, Physical Medicine & Rehab, Internal Medicine, and Psychiatry at -1.43, -0.86, -0.83, -0.75, and -0.72, respectively. Z scores closest to zero were found for Child Neurology at -0.02.

Research abstracts, presentations, and publications

**Figure 9:**
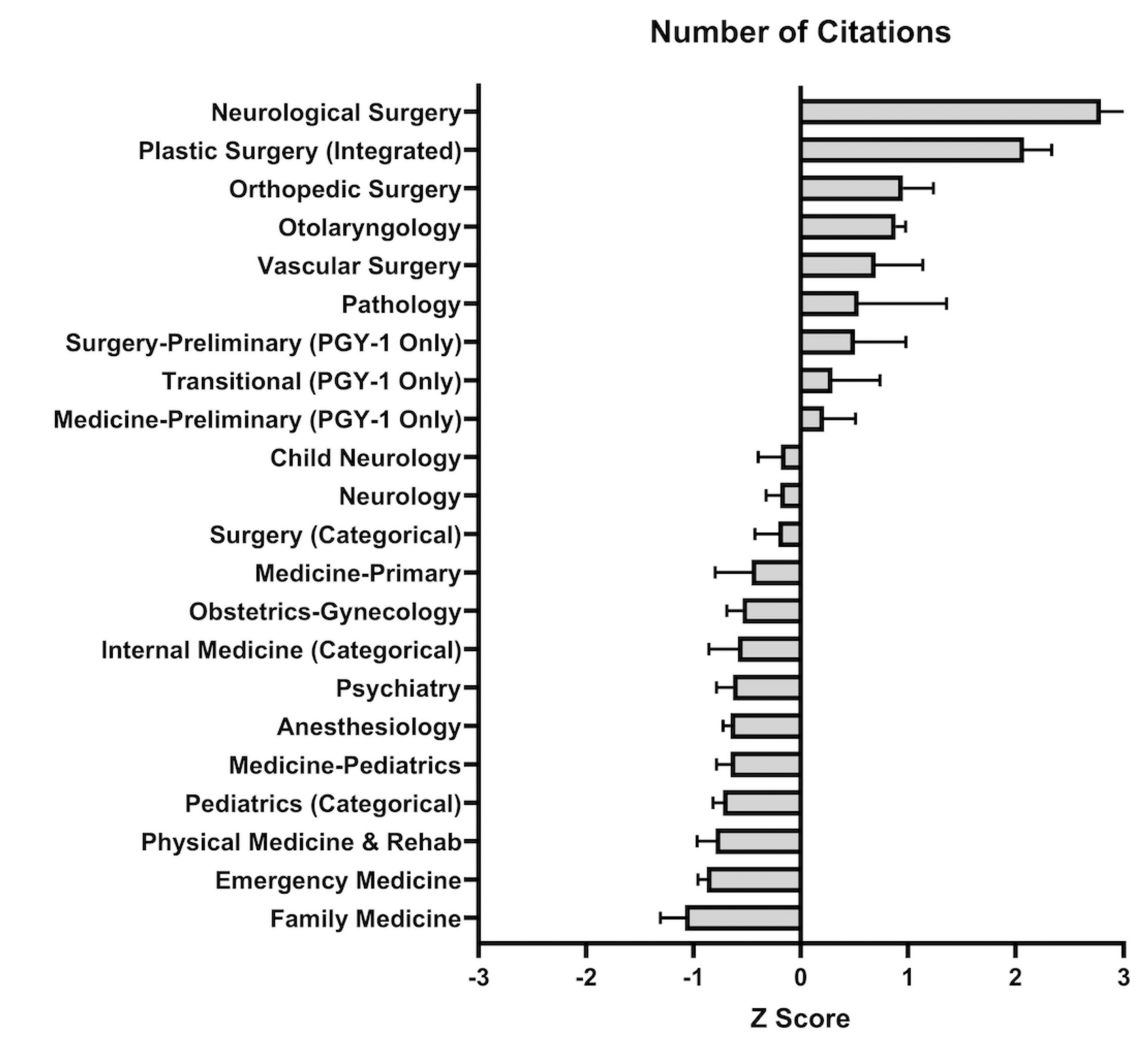
Matched Applicants’ Research Citations (Research Abstracts, Presentations, and Publications) by Specialty Matched applicants’ research citations were analyzed for each specialty. Average values and standard deviations were obtained from 2013 to 2020. Then, a Z score was calculated for each specialty. The top four specialties were Neurological Surgery, Plastic Surgery (Integrated), Orthopedic Surgery, and Otolaryngology. Of note, these specialties were the same top four as identified by the USMLE Step 1 Z score.

The Z score for each specialty in research abstracts, presentations, and publications was calculated (Figure 9).

The highest positive Z scores included Neurological Surgery, Plastic Surgery, Orthopedic Surgery, Otolaryngology, and Vascular Surgery at 2.80, 2.08, 0.96, 0.89, and 0.70, respectively. The highest negative Z scores included Family Medicine, Emergency Medicine, Physical Medicine & Rehab, Pediatrics, and Medicine-Pediatrics at -1.08, -0.87, -0.79, -0.73, and -0.65, respectively.

Comparison of overall characteristics

**Figure 10:**
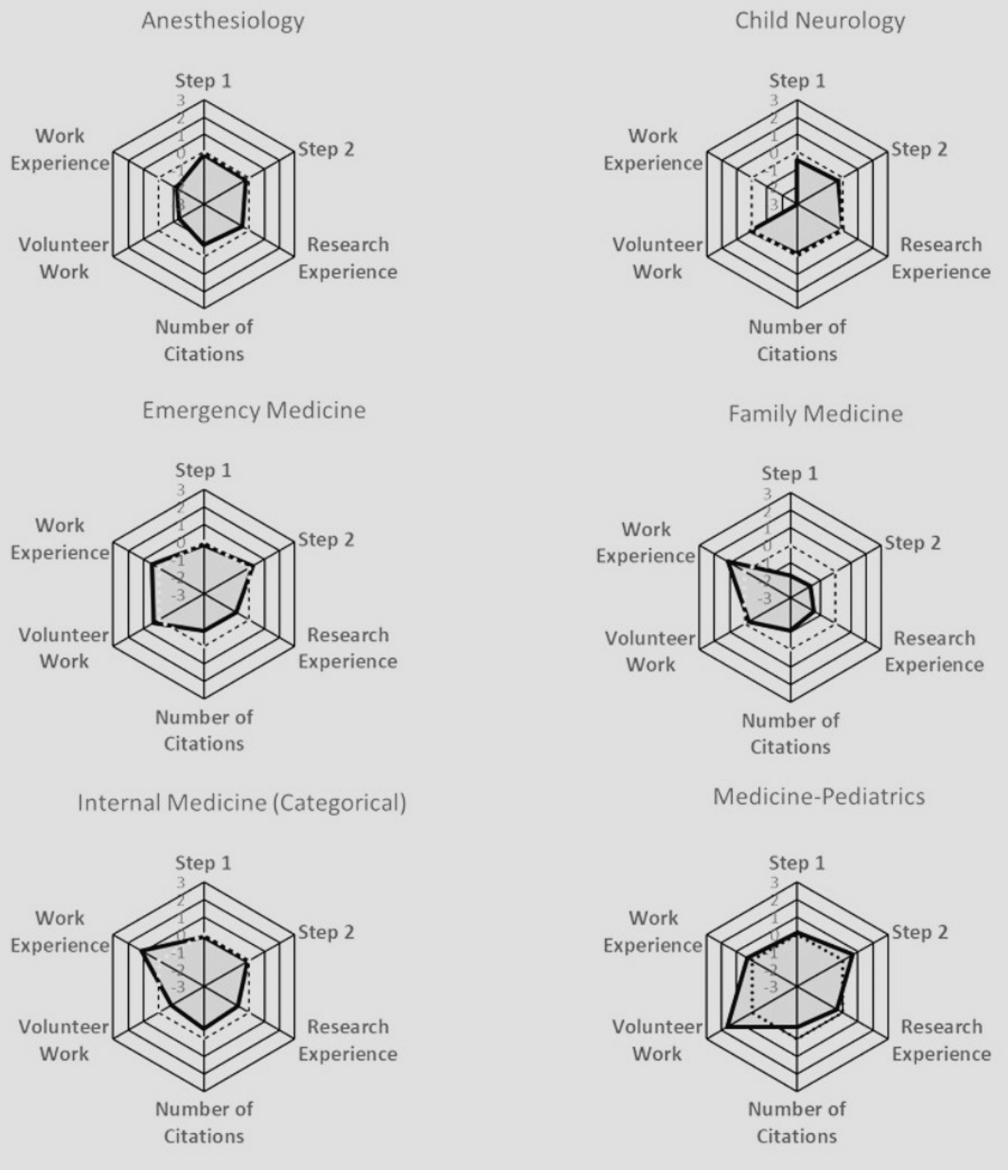
Radar Plots Comparing All Matched Applicants' Quantitative Characteristics for Anesthesiology, Child Neurology, Emergency Medicine, Family Medicine, Internal Medicine (Categorical), and Medicine-Pediatrics Radar plots of Z scores. Each specialty was characterized by a radar plot with six axes: United States Medical Licensing Examination (USMLE) Step 1 score, USMLE Step 2 score, number of Research Experiences, number of Citations, number of Volunteer Work experiences, and number of Work Experiences. The axis values ranged from -3 (the innermost) to +3 on the outside; the dotted line highlights the zero value. The shadowed area indicates the relative difficulty of each specialty.

**Figure 11:**
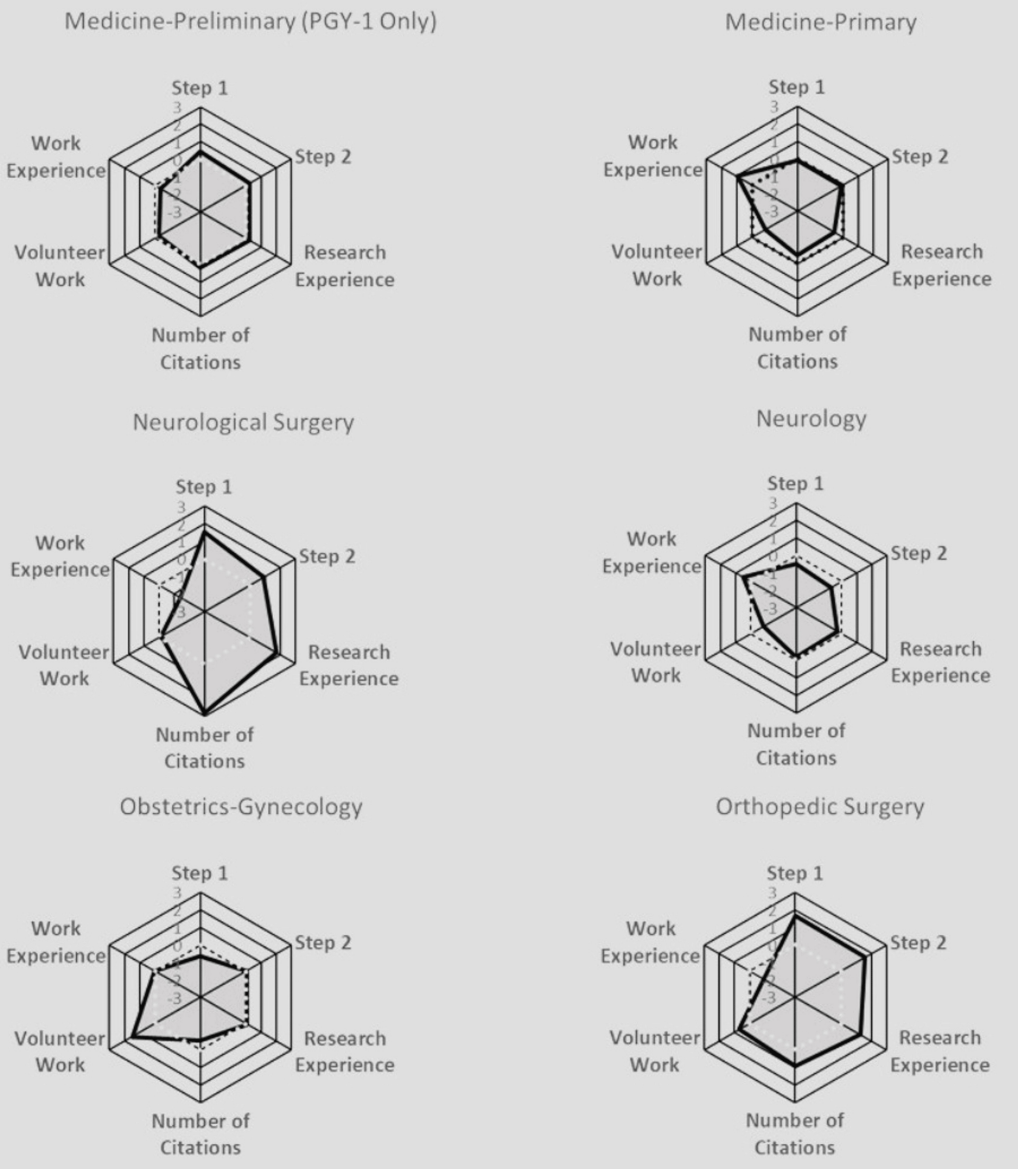
Radar Plots Comparing All Matched Applicants’ Quantitative Characteristics for Medicine-Preliminary, Medicine-Primary, Neurological Surgery, Neurology, Obstetrics-Gynecology, and Orthopedic Surgery Radar plots of Z scores. Each specialty was characterized by a radar plot with six axes: United States Medical Licensing Examination (USMLE) Step 1 score, USMLE Step 2 score, number of Research Experiences, number of Citations, number of Volunteer Work experiences, and number of Work Experiences. The axis values ranged from -3 (the innermost) to +3 on the outside; the dotted line highlights the zero value. The shadowed area indicates the relative difficulty of each specialty.

**Figure 12:**
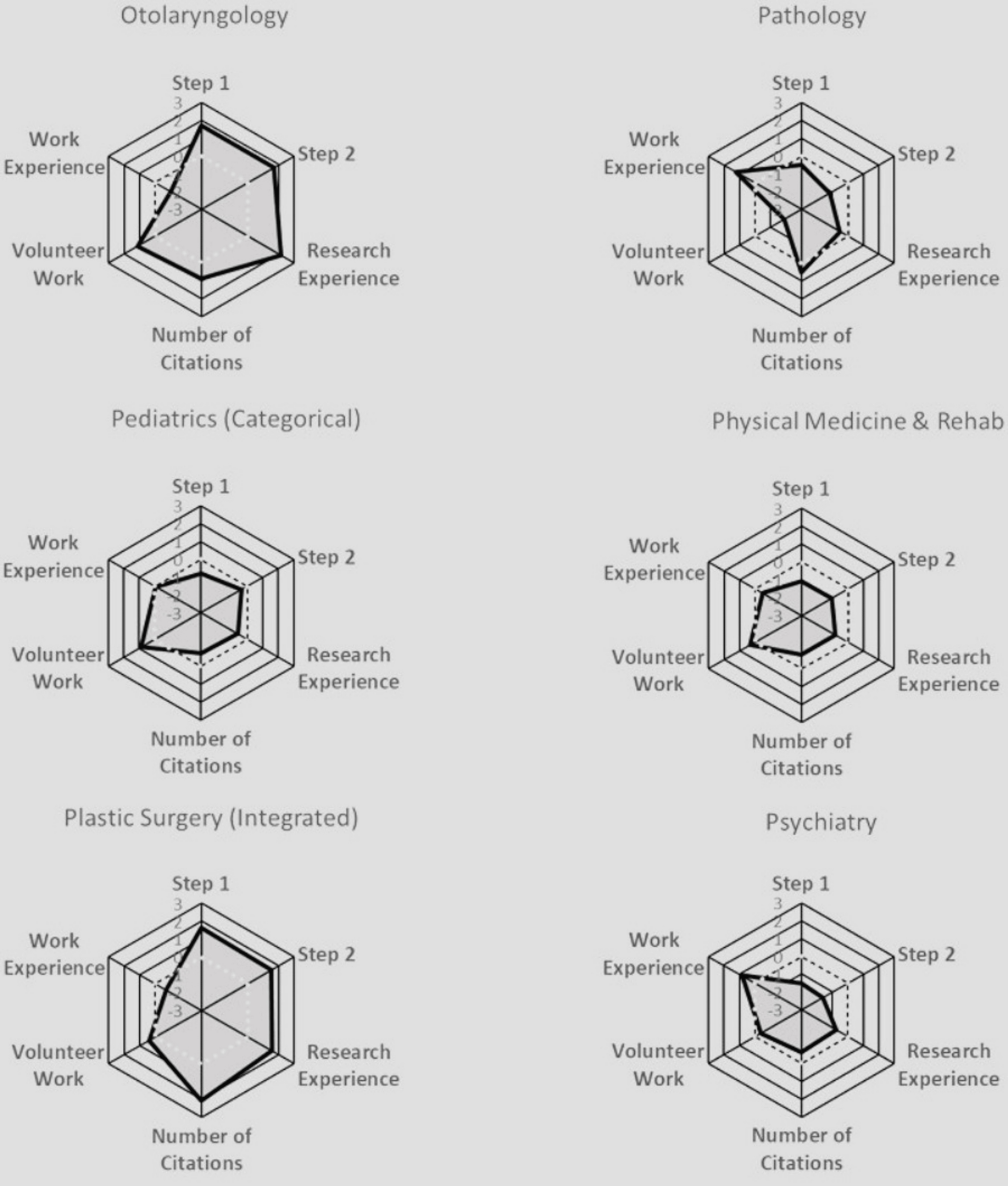
Radar Plots Comparing All Matched Applicants' Quantitative Characteristics for Otolaryngology, Pathology, Pediatrics (Categorical), Physical Medicine & Rehab, Plastic Surgery (Integrated), and Psychiatry Radar plots of Z scores. Each specialty was characterized by a radar plot with six axes: United States Medical Licensing Examination (USMLE) Step 1 score, USMLE Step 2 score, number of Research Experiences, number of Citations, number of Volunteer Work experiences, and number of Work Experiences. The axis values ranged from -3 (the innermost) to +3 on the outside; the dotted line highlights the zero value. The shadowed area indicates the relative difficulty of each specialty.

**Figure 13:**
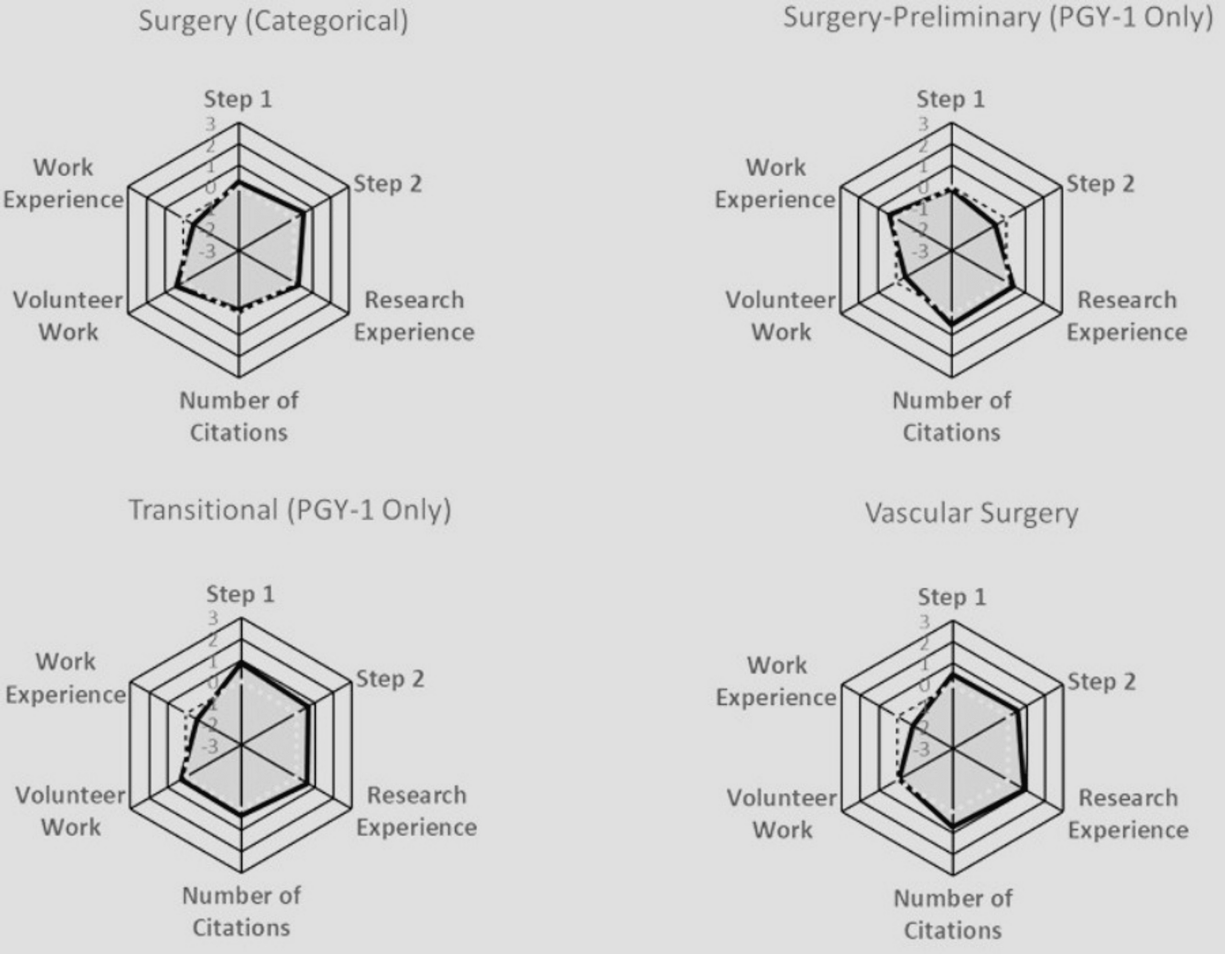
Radar Plots Comparing All Matched Applicants' Quantitative Characteristics for Surgery (Categorical), Surgery-Preliminary, Transitional, and Vascular Surgery Radar plots of Z scores. Each specialty was characterized by a radar plot with six axes: United States Medical Licensing Examination (USMLE) Step 1 score, USMLE Step 2 score, number of Research Experiences, number of Citations, number of Volunteer Work experiences, and number of Work Experiences. The axis values ranged from -3 (the innermost) to +3 on the outside; the dotted line highlights the zero value. The shadowed area indicates the relative difficulty of each specialty.

To compare all the quantitative characteristics together, including Step 1 score, Step 2 score, volunteer experience, work experience, research experience, and number of research abstracts, presentations, and publications, radar plots were used to compare these variables across specific specialties (Figures 10-13).

Anesthesiology prioritized all aspects except for work and volunteer experiences. Child Neurology exhibited no significant variations across all characteristics. Emergency Medicine leaned towards valuing volunteer and work experiences. Family Medicine placed a strong emphasis on work experience. Internal Medicine also heavily favored work experience. Medicine-Pediatrics leaned towards valuing volunteer experience. Medicine-Preliminary did not exhibit notable distinctions among all characteristics. Medicine-Primary leaned heavily towards work experience. Neurological Surgery leaned towards considering the number of research abstracts, presentations, publications, and research experience. Neurology showed a preference for work experience. Obstetrics-Gynecology leaned towards valuing volunteer experience. Orthopedic Surgery gave weight to Step 1 and Step 2 scores, with minimal emphasis on work experience. Otolaryngology prioritized research experience, Step 1, and Step 2 scores. Pathology emphasized work experience. Pediatrics favored volunteer experience. Physical Medicine & Rehab leaned towards valuing volunteer experience. Plastic Surgery prioritized the number of research abstracts, presentations, and publications, Step 1 and Step 2 scores, and research experience, over work and volunteer experiences. Psychiatry heavily values work experience. Surgery leaned towards all characteristics except for work experience. Surgery-Preliminary leaned towards considering work experience, the number of research abstracts, presentations, and publications, research experience, Step 1 score more than Step 2 score, and volunteer experience. Vascular Surgery leaned towards considering all characteristics equally, except for work experience.

## Discussion

This study sought to assess the relative importance of different quantitative variables on residency match rates and found that research abstracts, presentations, and publications were most strongly associated with a successful match. The association of the USMLE Step 1 and Step 2 scores remained consistent over the past 10 years, as shown by Figure 3, although this will likely change with USMLE Step 1 becoming a pass/fail test [10]. The number of research experiences had the greatest association with matching into Otolaryngology, Neurological Surgery, and Integrated Plastic Surgery (Z scores: 2.16, 1.73, and 1.54, respectively), while the number of research abstracts, presentations, and publications had the greatest association with matching into Neurological Surgery, Integrated Plastic Surgery, and Orthopedic Surgery (Z scores: 2.80, 2.08, and 0.96, respectively), as shown by Figures 8-9. These trends are similar to those historically observed for individual subspecialties, as shown in other databases such as the National Resident Matching Program (NRMP), which demonstrates that step scores and research productivity have the strongest association with successful matching [11].

The findings from this study demonstrate that the number of research abstracts, presentations, and publications had the greatest overall association with residency matching. The importance of research, specifically, has been well documented in terms of its association with an applicant’s chances of matching in certain competitive specialties [12]. For example, within Ophthalmology, applicants who had more impactful research, as assessed by Hirsch's index (h-index), had a higher chance of matching into a higher-tiered research institution [13]. Due to the significant impact of research on the application process, many applicants choose to take dedicated research time; however, a 2020 study found that this did not increase the chance of IMGs matching into general surgery residency, and program directors advised against it [14]. On the other hand, for specialties such as Otolaryngology, Plastic Surgery, or Neurological Surgery, dedicated research time may increase the odds of certain applicants matching into competitive residency programs [15-17]. Over the past decade, research has become more heavily emphasized for residency applications, likely due to the removal of some of the more objective metrics (e.g., numerical grading systems and standardized test scores) that medical schools had in place in the past. There was also a significant increase in research productivity around 2020, which may be related to the COVID-19 pandemic restricting volunteer and work experiences, thus encouraging more students to prioritize research. Furthermore, research productivity as a trainee has a direct impact on research performance as an attending, thus encouraging physicians to find innovative ways to improve the current standard [18].

With the recent adjustment to make the USMLE Step 1 a pass-or-fail test, rather than a numeric score out of 300, for all medical students starting in 2022, there will be a further emphasis on other extracurriculars and USMLE Step 2 scores [19]. The USMLE Step 1 used to be one of the most important screening and selection tools for selection committees, as they compare hundreds and thousands of applicants for just a few residency spots [20]. The AAMC made this change to hopefully alleviate some of the stress on medical school students in the first two years of their training, allowing them to spend time on becoming more well-rounded students and future physicians [21]. Makhoul et al. believe this change will lead to a heavier emphasis on performance during clinical years and extracurricular activities that students participate in during medical school, such as volunteering, work, leadership, and research experiences [22]. Our study found that the USMLE Step 1 and Step 2 scores played a significant role in a student’s acceptance into competitive residency programs, as shown by Figure 4 and Figure 6. We suspect that the importance of the other metrics in a student’s application, specifically research abstracts, presentations, and publications, will play a larger role going forward.

In addition to standardized test scores, extracurricular activities, such as volunteer, work, and research experiences, also played a large role in increasing an applicant’s likelihood of matching into their selected specialty. Similar to the results found in our paper, Leschke and Hunt also found that an increased number of volunteer experiences was an independent predictor for matching into competitive specialties, such as Neurological Surgery [6]. On the other hand, studies have shown that having more total volunteering hours or experiences did not impact an applicant’s chances of matching into Dermatology [23]. Our study found that work experiences did not play as large a role in influencing an applicant's likelihood of matching as some of the other extracurriculars (Figure 7), which is paralleled in the literature as well; an increased number of work experiences does not seem to be associated with an increased probability of matching into a competitive specialty, as shown by the radar plots [6]. In Plastic Surgery and Neurological Surgery specifically, all categories had a significant association with matching other than work and volunteer experience (Figures 10-13). Finally, our study found that the number of unique research experiences had a slight impact on match likelihood; however, it seems that research productivity, rather than the number of unique research experiences, was more beneficial in optimizing an applicant’s chance of matching, as depicted in the radar plots (Figures 10-13). Overall, more competitive specialties tend to prioritize research experience and productivity more than volunteering and work experiences. This may lead to increased inequality in the overall application process, since many of the quantitative variables that residencies tend to value may require flexibility to take time off (e.g., a research year) or undertake an unpaid position, which may not be feasible for all medical students [24].

Limitations

Our findings can be viewed in light of the following limitations related to subjective variables considered within the match process. First, this study used aggregate and specialty-level data, which prevents the ability to infer individual-level match probabilities. Thus, these associations may not apply to individual applicants and introduce the risk of ecological fallacy. Second, we were unable to assess key metrics, such as LORs, away rotations, medical student performance evaluation (MSPE), personal statements, and interview performance, which are more subjective metrics that play a large role in the application process. Future directions of this project may include qualitative analysis with program directors to assess which metrics influence medical student acceptances and how this has changed since USMLE Step 1 became pass/fail. Third, the AAMC database did not report all of the values, including the percentage of women in certain specialties; thus, those values were not included in this analysis. Consequently, the absence of this data prevents an examination of equity-related considerations, and reliance on quantitative metrics may differentially affect underrepresented applicant groups. Finally, this study was retrospective in nature, and we were unable to definitively conclude whether these aspects of a student’s application directly impacted their likelihood of matching into residency or if there were other metrics that contributed more heavily. A prospective study focused on the impact of individual extracurricular factors, stratified based on the student’s specialty of choice, would also help better identify the effect of each extracurricular factor on a student’s chance of matching.

## Conclusions

Although there is a wide range of factors that impact the residency match process, this study focused on quantitative variables, as they are objective and the most consistently reported metrics available for analysis, while variables such as LORs are inherently subjective. Our study found that research abstracts, presentations, and publications, specifically, which are reflective of overall research productivity, have the greatest association with a student’s probability of matching to a residency position, regardless of the intended specialty. Quantitative variables related to research and standardized test scores had a greater association with matching into more competitive specialties. With the emphasis on holistic review for applications, our contributions suggest that students should get involved in activities they are passionate about early on, including research experiences, since subjective (qualitative) metrics, such as LORs and personal statements, will likely have a more significant impact in the match process going forward. The contributions from this study offer guidance for students to make informed decisions on how to utilize their time more efficiently and choose activities in alignment with their interests and intended specialty.
